# Fertility-Sparing and Less Radical Surgery for Cervical Cancer

**DOI:** 10.1007/s11912-022-01317-w

**Published:** 2022-08-12

**Authors:** Samantha H. Batman, Kathleen M. Schmeler

**Affiliations:** Department of Gynecologic Oncology and Reproductive Medicine, Unit 1362, The University of Texas MD Anderson Cancer Center, 1515 Holcombe Boulevard, Houston, TX 77030 USA

**Keywords:** Cervical cancer, Fertility-sparing, Conservative surgery, Conization, Simple hysterectomy, Simple trachelectomy

## Abstract

**Purpose of Review:**

Patients with early-stage cervical cancer who desire future fertility may be candidates for less radical surgery. We review the literature supporting this approach in early-stage disease.

**Recent Findings:**

Retrospective data have shown that in carefully selected patients, the risk of parametrial involvement is less than 1%. This has led to interest in moving away from radical surgery towards more conservative approaches. Data from the newly published ConCerv trial, a prospective study evaluating the feasibility of conservative surgery in women with early-stage, low-risk cervical carcinoma, suggest that conservative surgery is feasible and safe in this patient population. Furthermore, neoadjuvant chemotherapy is being assessed as an option to extend fertility-sparing treatment to a larger group of women.

**Summary:**

Less radical surgery may be appropriate for carefully selected women with early-stage, low-risk cervical cancer, including those desiring future fertility.

## Introduction

Radical hysterectomy, which includes removal of the uterus, cervix, parametria, and upper portion of the vagina, has been the standard of care for women with early-stage, surgically resectable cervical cancer [1]. However, radical surgery is associated with significant morbidity in up to 25% of patients [2] and these sequelae include long-term bladder dysfunction, lymphedema, bowel dysfunction, and infection [3]. Several retrospective studies have demonstrated that women with early-stage disease and favorable pathologic characteristics have a risk of parametrial involvement of less than 1% [4], bringing into question the utility of radical surgery in this cohort.

Through the advancement of diagnostic tools and public health screening programs, women are increasingly being diagnosed with cervical cancer at earlier stages and at a younger age [5]. The loss of fertility in women with a history of gynecologic malignancies has been shown to be associated with depression, grief, stress, and sexual dysfunction [6]. Meanwhile, delayed childbearing in the USA has resulted in an increase in the age at first birth, in part due to the shift in first births to those who are 35 years and older [7], thus highlighting the increasing need for fertility-sparing treatments that do not significantly affect prognosis.

In this context, the management of early-stage cervical cancer has become less straightforward, and must weigh patient desires surrounding childbearing, long-term sequelae of radical surgery, and achieving the most optimal prognosis. In this review, we outline the fertility-sparing treatment options for early-stage cervical cancer, particularly highlighting cervical conization, trachelectomy, and neoadjuvant chemotherapy, and review their associated oncologic outcomes.

## Options for Fertility-Sparing Treatment

### Radical Trachelectomy

Radical trachelectomy, which entails en bloc removal of the cervix, upper vagina, and parametria while leaving the uterine body and fundus in situ, has been a widely accepted fertility-sparing procedure since its development in 1987 by French surgeon Daniel Dargent [8]. It is the current standard of care for women with early-stage cervical cancer desiring fertility preservation. Different surgical approaches have been used including the classic Dargent radical vaginal trachelectomy with laparoscopic pelvic lymphadenectomy, abdominal trachelectomy, and total laparoscopic or robotic-assisted laparoscopic routes [9]. The ideal candidate for radical trachelectomy should be of reproductive age, have a low-risk histology (squamous, adenocarcinoma, adenosquamous), negative lymph nodes of imaging, and a small lesion (≤ 2 cm) [10]. Pelvic MRI is recommended to rule out upper endocervical involvement [10].

A prior study by Machida et al. demonstrated that tumor factors are a key component to successful fertility-sparing treatment via trachelectomy and proposed that ideal candidates were those with small tumors (≤ 2 cm) without evidence of nodal metastases or deep stromal invasion and were of lower-risk histologies [11]. The authors found that patients with these features were noted to have a 5-year recurrence rate of 2.8% following trachelectomy [11]. Of note, stage IB2 cervical cancer includes tumors that are > 2 cm and ≤ 4 cm. While patients in this cohort can be considered for fertility-sparing treatment per National Comprehensive Cancer Network (NCCN) guidelines, 20–44% of patients with stage IB2 disease have been noted in prior studies to require adjuvant treatment post-radical trachelectomy [8, 12].

While radical trachelectomy has been associated with a low rate of recurrence, it is associated with similar risks to radical hysterectomy including bladder dysfunction, lymphocele, hematoma, and lymphedema [13]. Past studies have shown that approximately 60% of radical trachelectomy specimens have no residual disease following a diagnostic cone or loop electrosurgical excision procedure (LEEP) [13]. Furthermore, obstetric and pregnancy outcomes have been known to be compromised depending on route of radical trachelectomy, with the literature reporting 60% of patients developed a complication from abdominal trachelectomy that affected their fertility [14]. Thus, surgeons have been looking to less radical forms of treatment for their carefully selected patients.

### Decreasing Radicality

As previously mentioned, radical surgery is associated with significant patient morbidity. In a systematic review of radical versus simple hysterectomy for early-stage cervical cancer, Wu et al. noted that the most commonly described complications were lymphedema (24%), lymphocysts (22%), and urinary incontinence (18.5%) [15]. Rates of blood transfusion around the time of radical hysterectomy have been reported to be as high as 77% [16]. Many of these complications are felt to be related to the parametrial dissection involved in these radical procedures. However, in a select group of patients, parametrectomy may not be necessary. In a study assessing parametrial involvement after radical hysterectomy in women with early-stage disease, Frumovitz et al. found that 0% of women with tumor size of 2 cm or less without lymphovascular space invasion (LVSI) had parametrial involvement, while 0.7% of patients had a positive pelvic lymph node with a negative parametrium [17]. Ultimately, the authors concluded that patients with squamous, adenocarcinoma, or adenosquamous lesions of any grade, with tumor size of 2 cm or less, and no LVSI represented a low-risk group that could be considered for conservative surgery with preservation of the parametrium [17]. These data demonstrate that fertility and obstetric outcomes can be preserved without significant impact on oncologic outcomes in a select group of carefully chosen patients.

### Cervical Conization and Simple Trachelectomy

A cone biopsy, which is defined as the excision of a cone- or wedge-shaped piece of the uterine cervix including the transformation zone, is accepted as treatment for stage IA1 disease without evidence of LVSI in women who desire fertility preservation provided there are negative margins [5, 18]. Outside of this cohort, the NCCN recommends pelvic lymph node assessment with lymphadenectomy in addition to the fertility-sparing procedure [18]. Although systematic lymphadenectomy is the standard of care at many treatment centers, it has been shown that the rate of lymph node metastases is 2.9% in patients with early-stage disease and no LVSI, and 0% in patients with either stage IA2 disease or stage IB disease with grade 1 tumors [19].

Results from the recently published ConCerv trial by Schmeler et al. demonstrated that conservative surgery in early-stage (IA2–IB1), low-risk (negative LVSI, negative cone margins) cervical cancer was associated with a 3.5% recurrence rate and a positive lymph node rate of 5% [20]. Of note, in this single-arm, international, prospective trial of 100 women, 44% underwent fertility-sparing surgery with conization followed by nodal assessment only (either sentinel lymph node biopsy or full pelvic lymphadenectomy depending on the participating institution’s guidelines) [20]. In this treatment arm, two patients were found to have positive lymph nodes and the risk of recurrence was 2.4% (1 of 42 patients). This recurrence occurred in the first year of the study in a patient who was found to have grade 2 squamous cell carcinoma with 13 mm of invasion and positive margins at the time of her initial cone biopsy and the inclusion criteria for the ConCerv study were subsequently amended to become more conservative and include a depth of invasion of ≤ 10 mm and negative cone margins with no further recurrences [20]. 

Simple trachelectomy is the removal of the uterine cervix without parametrial resection (thus, the difference from cervical conization is a matter of degree of excision), and is usually done in association with pelvic lymphadenectomy [5]. In 2020, Plante et al. performed a retrospective review of a prospective series of patients who underwent simple vaginal trachelectomies (42 patients) or large cold knife cone biopsies (8 patients) with early-stage cervical cancer [21]. In this combined group, the authors found a 5-year progression-free survival of 97.9% and overall survival of 97.6%, with only one recurrence and death (2%) occurring in a patient who underwent simple trachelectomy [21]. These findings are similar to a smaller retrospective review of 14 women who underwent simple vaginal trachelectomy with no recurrences [22]. Raju et al. in 2011 reviewed data from 66 women who either underwent simple vs. radical vaginal trachelectomy [23]. The authors similarly found no recurrences in the simple vaginal trachelectomy group after a median follow-up time of 96 months, and noted no residual disease in 53% of those who underwent simple vaginal trachelectomy compared with 29% of patients who underwent radical vaginal trachelectomy (the authors did not comment on whether this difference was statistically significant or due to higher risk patients in the radical trachelectomy group) [23].

Two ongoing trials will continue to address the impact of non-radical surgery on this patient population. GOG 278 is a large, prospective cohort study that aims to examine the changes in bladder, bowel, and sexual function among women who have undergone conservative treatment for early-stage cervical cancer, and includes a fertility preservation arm of women undergoing conization and pelvic lymphadenectomy [24]. Women with stage IAI (LVSI +) and stage IBI (< 2 cm) cervical cancer with < 10 mm of invasion on their diagnostic pathology are eligible. The SHAPE trial, while not focused on fertility preservation, similarly seeks to add to the growing body of evidence evaluating the overall safety of conservative surgery in this group of patients by assessing the oncologic outcomes of less radical procedures [25]. This randomized trial compares radical hysterectomy to simple hysterectomy for early-stage (IA2–IBI), low-risk (< 10 mm invasion on diagnostic path, < 50% on MRI) cervical cancer [26]. This study has completed accrual and the final results will be available in 2023. If the results of these two additional trials confirm the findings of the ConCerv study, it is possible that the standard of practice may shift towards more conservative surgery in carefully selected patients.

### Lymph Node Assessment

Previous studies have shown positive lymph node rates of 3 to 8% [9, 20, 21] despite the above-mentioned low-risk pathologic features. In patients with tumor size > 2 cm, the incidence of positive lymph nodes approached 33% [27]. Thus, pelvic lymph node sampling is an important component of fertility-sparing surgery and represents an important prognostic indicator.

Given the surgical morbidity associated with lymphadenectomy, several studies have investigated the use of sentinel lymph node biopsy at the time of surgery for early-stage cervical cancer [28, 29]. The NCCN also recommends consideration of sentinel lymph node mapping at the time of surgery for early-stage disease [18]. A 2014 systematic review of 67 studies found a pooled detection rate of 89.2% and a pooled sensitivity of 90%, suggesting sentinel lymph node mapping is an accurate method of lymph node sampling in carefully selected patients [28]. The ongoing PHENIX/CSEM 010 multicenter, randomized controlled trial is addressing this question for patients with early-stage cervical cancer by performing sentinel node biopsy in all enrolled patients at the start of their radical hysterectomy and randomizing patients into an experimental (sentinel nodes alone) vs. referent (pelvic lymphadenectomy) arm [30].

### Minimally Invasive Surgery

The results of the recently published multicenter randomized Laparoscopic Approach to Cervical Cancer (LACC) trial demonstrating poorer oncologic outcomes using a minimally invasive approach in the setting of radical hysterectomy [31] call into question the use of a minimally invasive approach for radical trachelectomy. These results were further supported by the SUCCOR study, a European retrospective cohort study evaluating radical hysterectomy by open or minimally invasive surgery for stage IBI cervical cancer. The authors found an increased risk of recurrence (hazard ratio 2.07, *p* = 0.001) and increased risk of death (hazard ratio 2.45, *p* = 0.005) in the minimally invasive group as compared to the open group [32]. Interestingly, in a weighted cohort analysis, the SUCCOR study found a negative impact of the use of uterine manipulators on disease-free survival, particularly in patients with a tumor size greater than 2 cm. The authors report a disease-free survival at 4.5 years of 73% in the uterine manipulator group vs. 83% in the group without uterine manipulators (*p* = 0.0001). Furthermore, patients that underwent minimally invasive surgery without the use of a uterine manipulator had similar relapse rates to those who underwent open surgery [32]. The authors also evaluated the use of a protective vaginal closure over the tumor to decrease tumor spread at the time of the colpotomy. They found that those who underwent minimally invasive surgery with this protective technique had longer disease-free survival than those who had not (93% vs. 74% 4.5-year DFS, *p* < 0.001) and that patients who had the protective closure had similar relapse rates to those who were treated with open surgery [32].

The retrospective International Radical Trachelectomy Assessment (IRTA) study sought to address this question in the fertility-sparing setting by comparing disease-free survival in patients with stage IA2 or IB1 cervical cancer who underwent open vs. minimally invasive radical trachelectomy [33]. The study reviewed 646 eligible patients, who had squamous carcinoma, adenocarcinoma, or adenosquamous carcinoma and tumor size ≤ 2 cm and underwent either open or minimally invasive (robotic or laparoscopic) radical trachelectomy with nodal assessment (either full lymphadenectomy and/or sentinel node biopsy) [34]. The authors found no statistically significant difference (*p* = 0.37) in 4.5-year disease-free survival rates between the two groups, though they note that this may reflect the small number of recurrences in each group (4.7% in the open surgery group, 6.2% in the minimally invasive surgery group, *p* = 0.40) [34].

### Obstetrical Outcomes

Over two decades of experience with radical trachelectomy has confirmed a low rate of recurrent disease as well as reasonable obstetrical results. A 2011 study by Plante et al. reviewed data from 125 vaginal radical trachelectomies with a mean follow-up of 93 months and found 6 recurrences (4.8%) and 2 deaths (1.6%) [35]. In this cohort, 58 women conceived 106 total pregnancies. Seventy-seven of the pregnancies (73%) reached the third trimester and 58 of these (75%) delivered a term infant. Fifteen patients (13.5%) experienced fertility problems, 40% of which were due to cervical factor such as cervical stenosis [35]. A 2020 systematic review by Smith et al. assessing all routes of radical trachelectomy found a post-trachelectomy pregnancy rate of 23.9%, highest in the vaginal group [9]. Of these pregnancies, 75.1% resulted in live births and 39.6% of these were term deliveries [9].

Women undergoing less radical surgery for early-stage cervical cancer have been shown to have generally favorable fertility and obstetric outcomes as well. The 2020 Plante et al. study of simple vaginal trachelectomy/conization similarly showed promising results, with a total of 40 pregnancies that included five first trimester losses and one second trimester loss [21]. Three pregnancies resulted in mild prematurity (delivery between 34 and 36 weeks), while 75% delivered beyond 36 weeks gestation [21]. These results are similar to those found by Raju et al. who found a pregnancy rate of 80% among women who underwent a simple vaginal trachelectomy, all of which were term deliveries [23].

A systematic review by Bentivegna et al. confirmed that of those patients who had undergone a fertility-sparing procedure and desired pregnancy, over 50% achieved at least one pregnancy [36]. The majority of these pregnancies resulted in a live birth, with a reported live birth rate of approximately 70% [37]. However, there are elevated risks of preterm labor and birth with a reported prematurity rate of 38%, although these rates are noted to be lower in women who underwent simple trachelectomy, conization, or neoadjuvant chemotherapy followed by fertility-sparing surgery as compared to other conservative procedures [37]. Most commonly, the risk of premature delivery is secondary to premature rupture of membranes [37].

### Neoadjuvant Chemotherapy Followed by Surgical Resection

Many women desiring fertility preservation have cervical tumors that do not meet the existing criteria for a fertility-sparing approach. Neoadjuvant chemotherapy has been used in this setting is to reduce tumor size to facilitate surgical resection. This has been studied previously in women with larger tumors > 2 cm who desired future fertility and was shown to reduce nodal metastases, parametrial invasion, and tumor size [5, 38]. In 2011, Fokom Domgue et al. performed a literature review of 47 cases of stages IBI–IIA cervical cancer and calculated a crude recurrence rate of 8.5% and a crude mortality rate of 2.1% using this approach [5]. The authors then reviewed obstetrical outcomes from these studies and concluded that the live birth rate was 78.6% [5].

While neoadjuvant chemotherapy is accepted as a treatment option for tumor sizes between 2 and 4 cm, there is less data regarding its role in tumors < 2 cm. A 2021 systematic review by Noll et al. sought to compare oncologic and obstetric outcomes for patients who received neoadjuvant chemotherapy before cervical conization versus upfront conization in patients with a tumor size < 2 cm [39]. They reviewed data from 261 patients with a median follow-up time of 63.5 months and found no difference in disease-free survival or overall survival between the two groups. They did note, however, that patients who underwent upfront cervical conization had a higher rate of fertility preservation (99.1%) compared to an 81.4% fertility preservation rate in the neoadjuvant chemotherapy group [39]. There were no statistically significant differences in live birth rate or pregnancy loss [39].

The optimal timing of nodal assessment for patients undergoing neoadjuvant chemotherapy is unknown. Vercellino et al. evaluated a cohort of women who underwent neoadjuvant chemotherapy and surgery and concluded that nodal assessment prior to therapy can help identify a high-risk group who should avoid fertility-sparing treatment given the elevated risk of recurrence [27]. However, they also note that based on the findings from Slama et al. that demonstrated that use of neoadjuvant chemotherapy significantly reduced the prevalence of metastatic involvement of sentinel nodes [40], neoadjuvant chemotherapy prior to lymphadenectomy may have led to the operability of six additional women [27].

An ongoing multicenter, prospective, single-arm study CONTESSA/NEOCON-F is assessing the feasibility of preserving fertility in women with node negative stage IB2 cervical cancer with the use of neoadjuvant chemotherapy followed by fertility-sparing surgery [41]. Patients must have tumors measuring 2–4 cm and negative pelvic nodes. They undergo three planned cycles of neoadjuvant chemotherapy with a platinum-based agent in combination with paclitaxel and if a complete or partial response (< 2 cm residual lesion) is noted, they undergo cervical conization or simple trachelectomy at the providers’ discretion [41].

## Future Directions

As discussed above, there remain several unanswered questions as the trend continues to shift towards less radical surgery for early-stage, low-risk cervical cancer. Table 1 summarizes the ongoing clinical trials in this area. Among these are questions regarding the optimal timing and technique for lymph node assessment in this population. The issue of sentinel node biopsy over full lymphadenectomy for early-stage disease is being addressed by the currently enrolling PHENIX trial [30]. Neoadjuvant chemotherapy is being explored as a way to offer fertility-sparing treatment to a larger cohort of women by including those with tumors between 2 and 4 cm, and is being address by the ongoing CONTESSA trial [41], though the optimal timing of lymph node assessment in relation to neoadjuvant chemotherapy remains unknown. The GOG 278 and SHAPE trials are continuing to address the impact of non-radical surgery on carefully selected patients with early-stage, low-risk cervical cancer and may add to the findings of ConCerv, which demonstrate the safety of conservative surgery in this group [20, 24, 25]. In light of the recent LACC trial [31] findings, there is also concern regarding the safety of minimally invasive approaches to fertility-sparing surgery. While the recently published IRTA study suggests no difference in overall survival rates between the open and minimally invasive groups, this may have been impacted by the overall low rates of recurrence and the retrospective study design. Further study into this area may shed more light on the oncologic safety of minimally invasive surgery in this setting. These ongoing trials will hopefully further strengthen providers’ ability to safely offer fertility-sparing surgery to carefully selected patients.

**Table 1:** Ongoing clinical trials for conservative surgery for early-stage, low-risk cervical cancer

Trial name	Objective	Trial design	Inclusion criteria	Study arms	Status
ConCerv (NCT01048853) [20]	Evaluate the feasibility of conservative surgery in women with early-stage, low-risk cervical cancer	Prospective, single-arm, multicenter	• FIGO 2009 stages IA2–IB1 cervical carcinoma • Squamous or adenocarcinoma histology • Tumor size ≤ 2 cm • No LVSI • Depth of invasion ≤ 10 mm • Negative imaging for metastatic disease • Negative cone margins	• Conization followed by nodal assessment (fertility preservation) • Conization followed by simple hyst + nodal assessment • “Inadvertent” simple hyst + nodal dissection	Completed and published
GOG 278 (NCT01649089) [42]	Study the physical function and quality-of-life before and after surgery in patients with stage I cervical cancer	Prospective cohort	• Stages IA1–IB1 disease • Squamous, adenocarcinoma, or adenosquamous histology • Tumor size ≤ 2 cm • Depth of invasion on diagnostic path ≤ 10 mm • No evidence of metastasis on imaging	• Cone biopsy and pelvic lymphadenectomy • Simple hysterectomy and pelvic lymphadenectomy	Recruiting
SHAPE (NCT01658930) [25]	Compare pelvic relapse-free survival in patients undergoing simple vs. radical hysterectomy	Randomized clinical trial	• FIGO 2009 stages IA2–IB1 • Adenocarcinoma, squamous, or adenosquamous histology • Tumor size ≤ 2 cm • Depth of invasion ≤ 10 mm or < 50% on MRI • No desire for future fertility	• Radical hysterectomy + pelvic lymph node dissection • Simple hysterectomy + pelvic lymph node dissection	Completed enrollment
CONTESSA (NCT04016389) [41]	Evaluate the safety of neoadjuvant chemotherapy followed by fertility-sparing surgery	Multicenter, prospective, single-arm, phase II trial	• FIGO 2018 stage IB2 cervical cancer with lesions measuring 2–4 cm • Lesion size assessed by pelvic MRI and physical exam • Squamous, adenocarcinoma, and adenosquamous histology • LVSI allowed • Pelvic lymph node dissection ± sentinel lymph node mapping to exclude node-positive patients	• All participants will receive neoadjuvant platinum-based chemotherapy, followed by assessment for response • If responded, undergo trachelectomy • If failed to respond, receive adjuvant treatment with chemotherapy and radiotherapy or have a hysterectomy	Recruiting
IRTA [34]	Compare 4.5-year disease-free survival after open vs. minimally invasive radical trachelectomy	Multicenter, retrospective	• Squamous, adenocarcinoma, adenosquamous histology • Tumor size ≤ 2 cm • Underwent open or minimally invasive (robot or laparoscopic) radical trachelectomy with nodal assessment (pelvic lymphadenectomy and/or sentinel lymph node biopsy)	• Open radical trachelectomy • Minimally invasive radical trachelectomy (laparoscopic or robotic)	Completed and published
PHENIX/CSEM 010 (NCT02642471) [30]	Compare oncological outcomes of sentinel lymph node biopsy with pelvic lymphadenectomy in patients with and without sentinel node metastasis	Multicenter, randomized control trial	• Patient age 18–65 years • Histologically confirmed, untreated stages IA1–IB2 cervical squamous carcinoma, adenocarcinoma, or adenosquamous carcinoma	• All patients will undergo sentinel node biopsy at the start of surgery, which will be sent for frozen section, and then patients are triaged to PHENIX-1 (node negative) or PHENIX-II (node positive) • Within each cohort, randomized in 1:1 ratio of sentinel nodes alone or pelvic lymphadenectomy • Radical hysterectomy performed for all patients	Recruiting

## Conclusions

Radical surgery has long been the standard of care for early-stage cervical cancer, despite significant known morbidity. As the average age of childbearing in the USA continues to increase and there is greater need for fertility-sparing procedures, the need for radical surgery has been called in to question. As reviewed, several studies have shown that in carefully selected patients, non-radical surgery can be performed without sacrificing oncologic or obstetric outcomes. These patients are typically those with small tumors (≤ 2 cm), low-risk histology (squamous, adenocarcinoma, adenosquamous), and no LVSI. In this group, the risk of parametrial invasion is very low. While less radical surgery is promising for this group, there remain several unanswered questions including use of neoadjuvant chemotherapy prior to surgery for larger tumors, timing and method of lymph node sampling, and use of minimally invasive surgery for this fertility-seeking population. Ongoing studies seek to address these unanswered questions to better inform our care of this patient population.
